# Effects of throat sizing and gasification agents in a biomass downdraft gasifier: towards CO_2_-free syngas production

**DOI:** 10.1039/d3ra01408h

**Published:** 2023-04-04

**Authors:** Ahmed M. Salem, Manosh C. Paul

**Affiliations:** a School of Engineering and Physical Sciences, Heriot-Watt University Edinburgh EH14 4AS UK; b Systems, Power & Energy Research Division, James Watt School of Engineering, University of Glasgow Glasgow G12 8QQ UK Manosh.Paul@glasgow.ac.uk; c Mechanical Power Department, Faculty of Engineering, Tanta University Tanta 31521 Egypt Ahmed_salem@f-eng.tanta.edu.eg

## Abstract

The gasification process in a downdraft biomass gasifier is investigated using Computational Fluid Dynamics (CFD). The aim is to develop a novel approach to reduce CO_2_ emissions from producer syngas while increasing the higher heating value (HHV). To this end, the effects of varying the throat diameter of the gasifier and gasifying media (air and oxygen) on the performance of gasification are investigated. The results reveal that as the throat ratio decreases for oxy-gasification, more CO, H_2_, and CH_4_ are produced, thus resulting in a HHV of 12.1 MJ Nm^−3^. For the same working conditions (ER, MC, and feedstock), the suggested design/optimum throat ratio of 0.14 is found to reduce CO_2_ by ∼55% compared to any other higher throat ratios, while simultaneously increasing HHV by ∼20% for both air and oxy-gasification cases. Additionally, the suggested throat ratio increases the gasification efficiency, carbon conversion and producer gas yield by 19%, 33%, and 22% respectively. Therefore, it shows a significant potential for CO_2_-free syngas production in the gasification process, demonstrating a promising technique that does not require any solvents, catalysts, absorbers, or additional CO_2_ removal. Lower throat ratios further favour the higher yield of syngas, HHV, gasification and conversion efficiencies, with better gasifier performance.

## Abstract 2

The influence of throat diameter and gasifying medium on gasifier performance is investigated using a CFD model. Lower throat ratios favour higher gasification efficiency, carbon conversion, and producer gas yield, with potential to reduce CO_2_ emissions by 50%.

## Graphical abstract

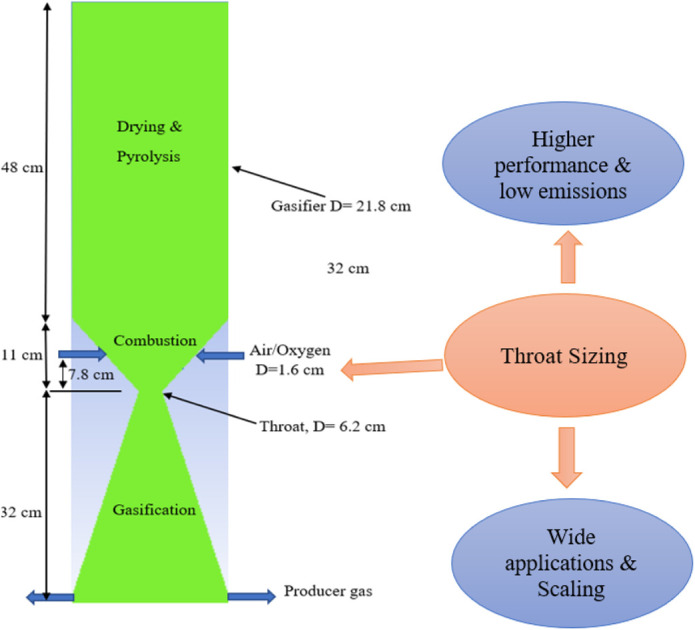

## 1. Introduction

The gradual use of fossil fuels for energy production is escalating the negative impacts on the environment and climate change due to CO_2_ production.^1–3^ The increased rate of depletion of fossil fuels and the worlds' increased energy demands are all leading to the focus on renewable energy sources. Biomass is a renewable and sustainable resource for energy and has CO_2_ neutrality. Energy recovery from biomass could be done through combustion, pyrolysis, and gasification.^4–6^ One of the most promising ways for energy production from biomass is gasification. It is estimated that 10% of energy production around the world is met from biomass.^7,8^

Designing a gasifier requires complicated steps and considers different aspects *e.g.*, required thermal power, as well as biomass type, size, moisture, and ash content. As a result, it requires a time consuming experiment or a detailed numerical modelling which proves its ability in the gasification process simulation and design.^9,11^ Although experiments are effective and reliable in designing a gasifier, it is a costly, sometime risky and also time consuming. Consequently, researchers are using modelling to simulate and predict gasifiers behaviour. Different modelling tools are used in the gasification process varying from equilibrium^12,13^ to kinetic,^11,14–17^ and Computational Fluid Dynamics (CFD).^10,18–20^

Equilibrium^12,13,21,22^ and kineti^15–17,23,24^ models are widely used in pyrolysis and gasification of biomass. However, there are some limitations which restrict the applicability of both the kinetic and equilibrium models. For example, gasifier design is a complex process affecting the production of syngas and tar content. Kinetic models can only address chemical reactions and rates which do not depend on the gasifier geometry. A robust modelling tool should also consider multiphase fluid dynamics, heat and mass transfer, and chemical transport. The solid and gas phase reactions and their interactions cannot be covered through kinetic models.^9,25^ To address all these factors, CFD modelling techniques are strongly recommended.^9,18^

CFD models are widely used in the process of gasification influenced with different chemical kinetics, and rates of reactions. The approaches of variations are based on the gasifier geometry, design, feedstock, operating parameters, and gasifying agent. Using the appropriate modelling techniques, CFD models are expected to reduce the time to design a gasifier and predict gasification output of each experiment based on a specific feedstock and working parameters.^26^ As a result, CFD models are emerging as an effective method in the gasification process simulation for different gasifier types.^26–28^

L. Yu *et al.*^29^ introduced a numerical model for coal gasification inside a fluidized bed gasifier. They combined Arrhenius rate reactions for coal gasification with a kinetic theory of granular flow (KTGF). After the validation of model against experimental data, it was then used to study the effect of changing gasifier height on the syngas composition, velocity, and temperature along the gasifier bed. Whereas a detailed model was built by Fletcher *et al.*^30^ using CFX4 package, for the gasification of biomass in an entrained flow gasifier. They used Lagrangian approach in modelling the particles entering the gasifier, followed by volatiles release and gasification. The concentrations of gas species are obtained by solving the transport equations and heterogeneous reactions. Producer gas composition with gasification temperature was presented at the gasifier outlet and found in a good agreement with experimental results.

The model built by Kumar and Paul,^10^ for a downdraft biomass gasifier used ANSYS Fluent software, and simulated a 2D, 20 kW downdraft gasifier. The four main gasification zones were included in the model by the Euler–Lagrangian discrete phase approach. The model was validated against the experimental data and kinetic model of ref. 31. Additionally, different feedstocks were used with different air equivalence ratio (ER) to study the model sensitivity on the gasification process. Although the model showed stable and reliable results, it could not perform better under ERs below 0.35. Furthermore, the model was converted to a 3D model using rubber wood as a feedstock.^18^ The 3D model found a good agreement with the previous experimental data at same working conditions.

More details about CFD modelling within different gasifiers could be found in ref. 32, 33, 34, 35 and 36. However, most of the previous works do not include oxy-gasification effect in CFD modelling, and its effect on the gasifier design and output. Hence, the main goal of the current research is to put more focus on the effect of air and oxy-gasification towards improving the yield of hydrogen enrich bio-syngas and how the gasification agent alternation further influences the key design parameter of a downdraft gasifier *i.e.*, the throat ratio (*e.g.*, throat/gasifier diameter). Consequently, their combined effects on the overall gasifier performance will be further examined and explained.

Couto *et al.*^35^ presented a 2D numerical model based on CFD framework along with experiments to study the effect of using oxygen enriched air on the process of biomass gasification. Eulerian–Eulerian approach was used in exchanging mass, energy, and momentum. The model was validated against their experimental data and found a good agreement. The influence of oxygen on steam to biomass ratio, syngas composition, and temperature along gasifier was examined. They found that N_2_ and H_2_ concentrations decrease as a function of oxygen content, while CO_2_ concentrations were found to increase. They used KTGF, DPM, and k-epsilon turbulent model in the simulation process. However, they did not argue over the use of pure oxygen on gasification performance and producer gas quality. Additionally, the study does not include any effect of gasifier design and geometry, as well as the corresponding impacts of using different oxidizers.

Furthermore, one of the key parameters during the design of a gasifier is the throat diameter. It has a great effect on the producer gas composition, gasifier power, and tar formation, as shown in the kinetic model study of.^31^ Some CFD studies focused on the effect of throat angle,^37,38^ while others studied the effects of number and angle of nozzles *e.g.* (ref. 39 and 40). However, few numerical and experimental studies mentioned the throat diameter effect on the gasification process. Prasertcharoensuk *et al.*^41^ numerically studied the optimization process of a 20 cm throat of a downdraft gasifier using ANSYS CFD. Producer gas composition and temperature distribution were examined for different throat diameters. The modelling results were validated against experimental results and found to have a good agreement. Maximum value of H_2_ was found to be 31.2 vol%, and H_2_/Co ratio was found to be 1.25 at a throat diameter of 0.4. They used the throat to gasifier diameter ratio varying from 0.25 to 0.5. However, the effect of reducing the throat/gasifier diameter below 0.25 was not examined.

On the other hand, an experimental study was carried out by Montuori *et al.*^42^ They studied the effect of the throat diameter sizing on gasifier performance, and the whole gasification plant stability was coupled with an internal combustion engine. The fixed bed gasifier performance was examined in conjunction with syngas production and electricity generation. Air was used as a gasifying medium with two throat diameters 7 and 10 cm. They reported that 10 cm throat diameter is the most convenient for syngas production (31% increment), with the plant electricity generation reaching 40%. While Gunarathne *et al.*^43^ experimentally examined the effect of changing three throat diameters (125 mm, 150 mm and 175 mm) on downdraft gasifier output. Gasifier performance was reported by studying the specific syngas production, conversion efficiency, and heating value. They concluded that changing throat diameter has no significant effect on the producer gas composition. The highest rate of gas production was observed at a throat diameter of 175 mm, with ER being 0.425. Although previous studies included the effect of throat ratio and nozzle's diameter/height *e.g.* ref. 44, 41 and 45, the effect of changing gasifying medium and throat ratio on gasifier performance and CO_2_ emissions has yet to be investigated. Additionally, all studies used air as gasifying medium, and the main effect was on enrich hydrogen production. Furthermore, throat ratios below 0.25 was not examined in any of the mentioned studies.

A gasification process produces gases (CO, CO_2_, CH_4_, H_2_, N_2_, H_2_O), tar, and solid residues. The amount of CO_2_ produced depends on the gasifier type, feedstock, working conditions, and gasifying medium. Depending on the gasifying medium, the CO_2_ mol% of producer gas from steam, air, oxygen, and CO_2_ gasification produce (12–30)%, (15–38)%, (10–48)%, and (5–15)% respectively.^46–48^ The US dep. Of Energy reported in 2018 that 64 commercial plants for CO_2_ removal/capture is associated with syngas production plants. The most widely used technologies for removal are absorption-based (∼60%), followed by cryogenics (18%), adsorbers (10%), and other technologies.^49^ However, such technologies are still developing and cost intensive. Hence, it is better to focus on eliminating the production of CO_2_ during the gasification process as possible and this research addresses it.

To the best of authors' knowledge, previous studies, as per the literature review presented above, do not adequately cover throat sizing and its relationship with gasification processes when combining with different gasifying mediums. Additionally, the impact of varying agents, particularly oxy and oxy–air, on the producer gas quality, yield, carbon conversion, and gasification efficiency, and the subsequent heating value is not fully explored. Furthermore, one of the major goals of this paper, which addresses a crucial knowledge gap in the field, is to investigate the effect of modifying throat ratio and gasifying agent on minimising carbon dioxide emissions while simultaneously boosting hydrogen yield.

## 2. CFD model description

**Fig. 1:**
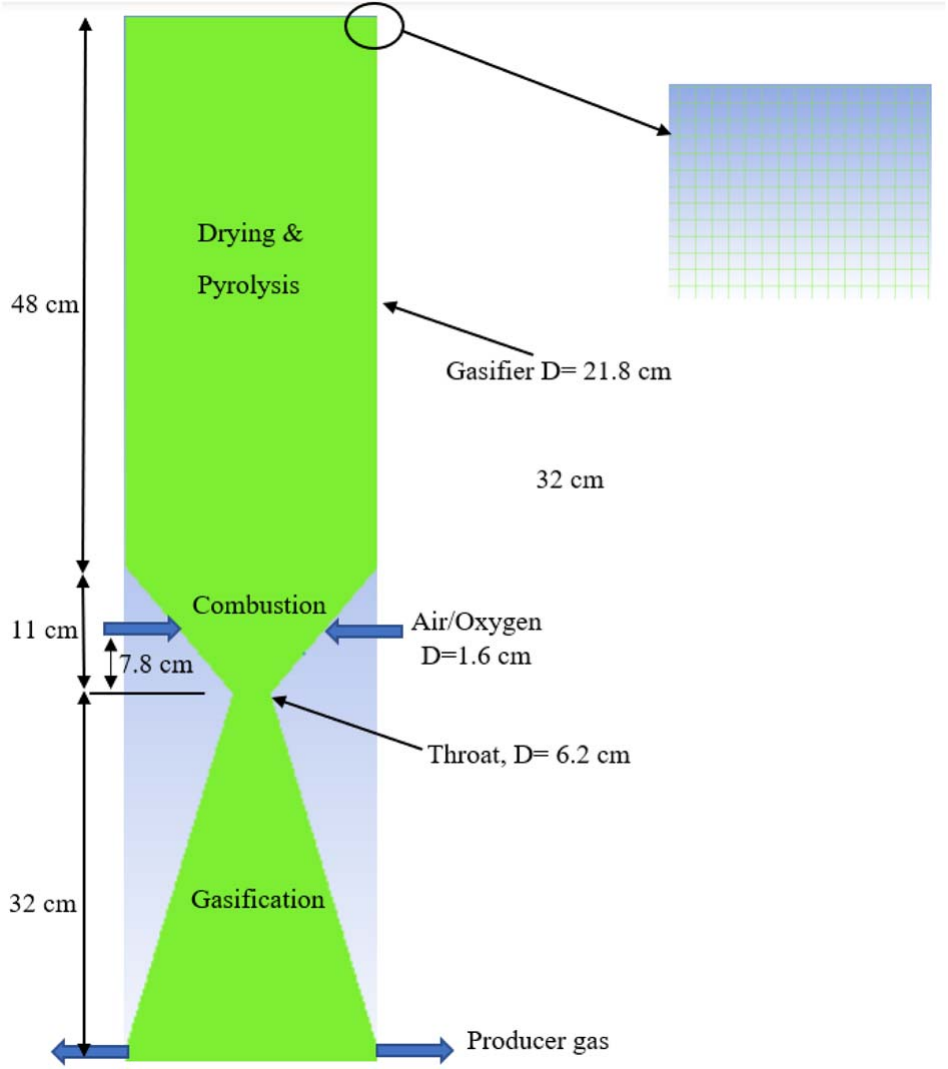
2D schematic of the proposed gasifier design.

The gasifier design is based on the kinetic model developed by the current authors^31^ in which a 20 kW downdraft biomass gasifier is modelled. The integrated model considers three zones – drying and pyrolysis, combustion, followed by gasification/reduction as illustrated in Fig. 1. Each zone is controlled by a set of detailed kinetic rate reactions used in ANSYS 19.0 (Tables 2 and 3). Further details for the gasifier schematic diagram in Fig. 1, and its dimensions are fully covered in ref. 9 and 31, and for brevity they are not repeated here.

**Table 1:** Feedstocks data used in validation and testing the model^18,57^

	Ultimate analysis db%					Proximate analysis db%			
	C	H	O	N	S	Vol.	FC	Ash	MC
Wood chips	54	6.0	40	0	0	70.0	20.0	0.338	7.36
Rubber wood	50.6	6.5	42	0.2	0.7	81.1	19.1	0.7	18.5

**Table 2:** Oxidation zone reactions

Reactions	*A* (1/s)	*E* (kJ mol^−1^)	Ref.
2C + O_2_ → 2CO	1.47 × 10^5^	112.99	58
2H_2_ + O_2_ → 2H_2_O	2.2 × 10^9^	109	59
CO + 0.5O_2_ → CO_2_	1.0 × 10^10^	126	59
CH_4_ + 2O_2_ → CO_2_ + 2H_2_O	4.4 × 10^11^	126	60

**Table 3:** Reduction zone reactions

Reactions	*A* (1/s)	*E* (kJ mol^−1^)	Ref.
C + CO_2_ → 2CO	8.268	188.2	58
0.5C + H_2_ → 0.5CH_4_	8.8894 × 10^−6^	67.16	58
C + H_2_O → CO + H_2_	42.5	142	58
CO + H_2_O → CO_2_ + H_2_	2.35 × 10^10^	288	61
CH_4_ + H_2_O → CO + 3H_2_	3 × 10^8^	125	59
CO_2_ + H_2_ → CO + H_2_O	1.785 × 10^12^	326	61

A zoomed in section from the top right-hand side of the gasifier is also presented in Fig. 1 to illustrate the structural mesh distribution created inside the gasifier. Air or oxygen is injected through the two nozzles at the gasifier sides within the combustion zone. The nozzles (*D* = 1.6 cm each) are specified at fixed height (7.8 cm) above the throat diameter based on the previous recommendations described in ref. 31. The feedstock is fed from top while producer gas is derived from bottom as showed in the figure. All the gasifier dimensions are illustrated in the figure based on the kinetic model predictions.^31^ The model assumes all the char is consumed during the reduction/gasification – the same assumption was made in the kinetic model.^31^ In addition, the model is considering the following assumptions:

• Steady-state simulations.

• Uniform spherical particle size.

• Tar and other higher hydrocarbons are neglected in the current model, for their complex formation and reaction rates.

• Char is fully consumed.

• All reactions take place under atmospheric pressure.

• Turbulence intensity and hydraulic diameter where specified for all inlets/exits for uniform distribution of flow inside the gasifier.

• Two equations k-epsilon model is specified for turbulence.

### 2.1. Governing equations

Species transport model is used along with the discrete ordinates (DO) radiation and k-epsilon turbulence models. Air and biomass are fed at 600 K, and 300 K respectively. The feedstock particles are modelled using a Lagrangian approach – discrete phase model (DPM). DPM considers the particles trajectories as a continuous phase of fluid in which an interaction between the particles takes place considering the mass and heat transfer equations. The conservation equations of mass, momentum, energy, and species transport are numerically solved under the turbulent flow steady-state condition with a set of finite rate kinetic reactions. These equations are presented as follows:^50,51^

Mass conservation:

1 ∇·(*ρv⃑*) = *S*_m_

Momentum conservation:

2 ∇·(*ρv⃑v⃑*) = −∇ (*τ̿*) + *ρg⃑* + *F⃑*

Energy conservation:3
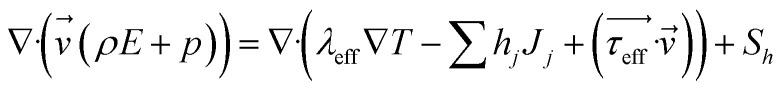


The turbulence k-epsilon RNG model is represented by4
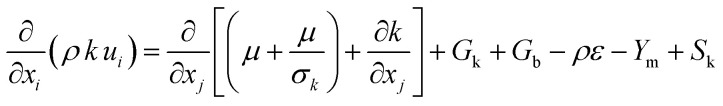
5
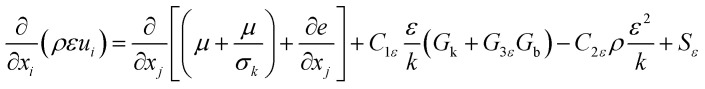
where the parameters *C*_1*ε*_ = 1.44, *C*_2*ε*_ = 1.92, *S*_k_ = *S*_e_ = 1, and *Y*_m_ = 0.09. *S*_m_ is the mass added to the phase (kg), *h*_*j*_ is the enthalpy of species (*j*), *τ̿* the stress tensor (pa), *λ*_eff_ is the effective conductivity, and *ε* is the turbulent dissipation rate (m^2^ s^−3^).

The species transport equation:^52^6
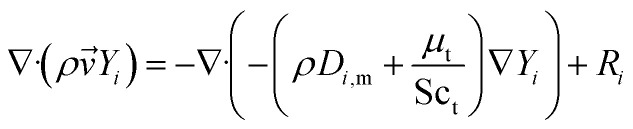
where *i* refers to different species in the simulation, Sc_t_ is the turbulent Schmidt number and is represented by the ratio of turbulent viscosity to eddy diffusivity, and *R*_*i*_ is the net rate of the production of different species (*i*) by the chemical reactions.

### 2.2. Devolatilization and biomass decomposition

Default drying model within the ANSYS directory^51^ is the Lee model^53^ which predicts the moisture evaporation and drying model for mixtures. It is applicable and shows good stability for the VOF multi-phase, and Euler–Lagrangian models. Consequently, it will be used in the current simulation.

The process of gasification is composed of four main steps. Drying, followed by pyrolysis and volatiles break-up, combustion, and gasification/reduction. The heat released during the combustion process drives the biomass drying and decomposition in the pyrolysis zone. After drying, the biomass first decomposes into volatiles and char, followed by further decomposition to form char and volatiles as illustrated by eqn (7) and (8).^54,55^

7 Biomass → volatiles + moisture + tar + char + ash

8 Volatiles → *x*_1_CO + *x*_2_CO_2_ + *x*_3_CH_4_ + *x*_4_H_2_

The volatiles are composed of gases (CO, CO_2_, H_2_, and CH_4_) and other HC components. The process of pyrolysis and biomass devolatilization starts after the drying process. Depending on its composition, biomass is decomposed into volatiles, char, tar, and ash. The model carries out an elemental mass balance for the volatiles to estimate its products. However, the CO concentrations are first calculated using the model proposed by^56^ which calculates the mass fraction of every species based on the pyrolysis temperature.

Eqn (8) describes the volatiles break-up based on the model proposed by.^56^ The model is further implemented inside the ANSYS directory to describe the species release during the pyrolysis process (eqn (7), and (8)) based on the ultimate analysis of the feedstock.

### 2.3. Boundary conditions

Two feedstocks are used in the current model for validation and studying the effect of varying the throat diameter on the gasifier performance and species behaviour.

### 2.4. Char and gas phase reactions

Tables 2 and 3 describe the different reactions used in the current model based on the recommendations of ref. 13 and 20, where *A* is the pre-exponential factor (1/s), and *E* is the activation energy in (kJ mol^−1^). The reactions represent the kinetic rate reaction data which take place in the oxidation and reduction zones. All the reactions are implemented inside the ANSYS code, including the volatiles decomposition reactions illustrated earlier.

### 2.5. Convergence criteria

The set of models and solution methods, and residuals control used are all concluded in Table 4.

**Table 4:** Solution methods followed in the CFD modelling

Phases	Euler–Lagrangian
Models included	Turbulence: k-epsilon 2 equations
	Species transport for finite rate/Eddy transport kinetic model
	Radiation: discrete ordinates
	Intensity and hydraulic diameter specification
Solution methods	Pressure–velocity coupling, coupled
	Pressure discretization scheme, PRESTO
	Momentum and energy; 2nd order upwind discretization scheme
Residuals level	10^−3^ for all variables, for energy and radiation 10^−6^

Two phase equations are solved numerically by an implicit finite volume method in ANSYS. A pressure–velocity coupling algorithm is used which solves the combined momentum and pressure-based equations.^51^ A spatial discretization for pressure is solved by PREssure STaggering Option (PRESTO) method which gives better accuracy and conversion for volume of fluids (VOF), and multi-phase modelling. Upwind scheme is used for solving the energy, momentum, and gas species discretization. Other boundary conditions are specified in Table 5.

**Table 5:** Boundary conditions used in the model

Inlet	Mass flow inlets for air nozzles and biomass feed
	Always supposed as normal to boundary
Outlet	Two exits for syngas zero-gauge pressure
	Back flow temperature was assumed 1000 K
Walls	Stationary walls
Turbulence	For assuring fully developed flows for air and biomass feeding, the turbulence is identified by the intensity and hydraulic diameter

## 3. Results and discussions

Following the mesh resolution study, the model is validated using data from a downdraft gasifier with the same design and working conditions. The effect of the throat/gasifier ratio on the producer gas heating value will be discussed, as well as process optimization. The results will be divided into two main categories; air gasification followed by oxy-gasification effects.

### 3.1. Mesh independency test

**Fig. 2:**
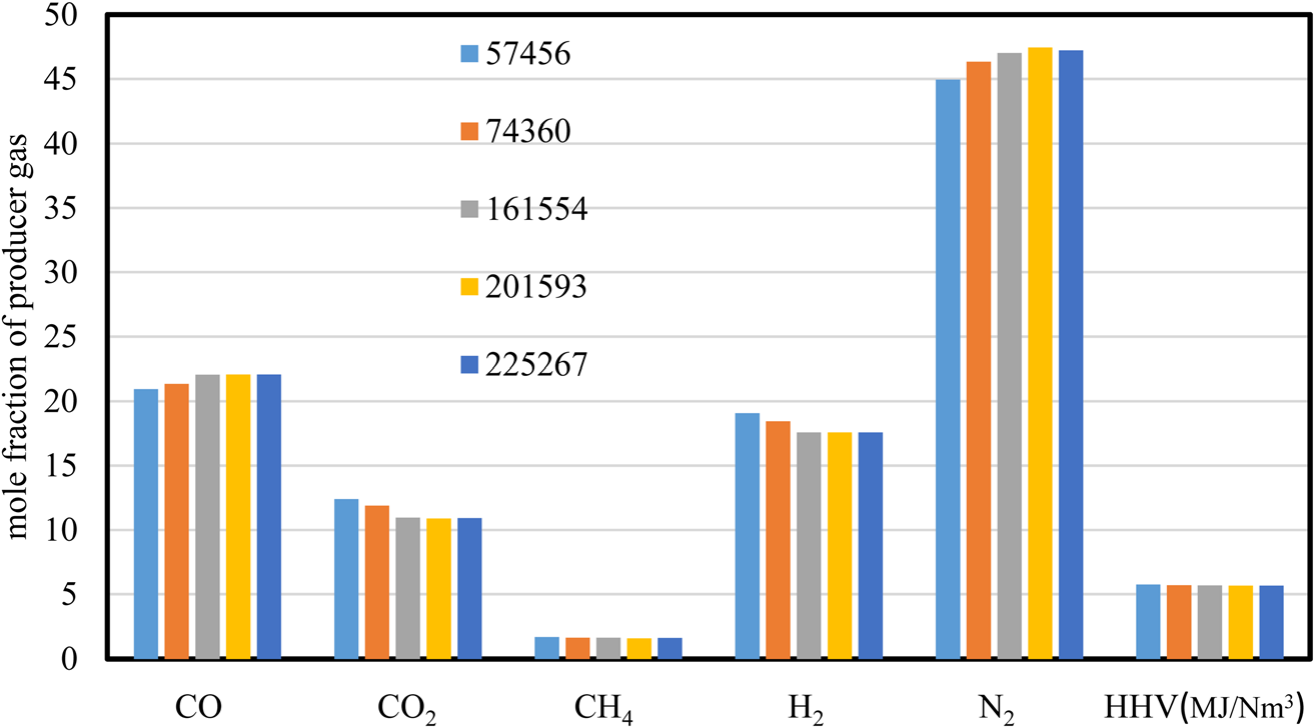
Producer gas composition at different cell numbers.

The mesh independency test is carried out using five different mesh sizes with cell numbers of 225 267, 201 593, 161 554, 74 360, and 57 456 respectively. The mole fraction of producer gas composition and its heating value are illustrated in Fig. 2, where air is used as a gasifying agent for wood chips gasification at ER of 0.3, and at a throat diameter of 8.8 cm.

The results of producer gas composition (mol%) and heating value (MJ Nm^−3^) for wood gasification showed slight variations in all the grid sizes used. The heating value of producer gas exhibits similar results with variances of less than 0.5%, demonstrating the consistency of the results throughout the five mesh sizes used. The mesh sizes higher than 74 360 cell numbers, show no variations in gas composition and heating value, implying stability of the results predicted. However, the higher grid size is a time intensive process and that requires higher computational cost. As a result, the mesh size of 74 360 is selected for the rest of the simulations carried out in this study.

### 3.2. Model validation

Besides the mesh independency test, which proves the model's stability, validation against experimental results^57^ is performed. The validation is carried out with the same feedstock (wood chips), ER (0.35), gasifying agent (air), and gasifier design (Tables 1 and 6). Additionally, rubber wood gasification is used as second feedstock and the results are compared with experimental data,^15^ and kinetic model results.^31^

**Table 6:** Gasifier design for current model and experimental data for validation

Gasifier design	Current model	Experiment^57^
Height, cm	90	91.7
External diameter, cm	21.8	21.9
Throat diameter, cm	8.8	8.8
Throat/gasifier D ratio, *r*	0.4	0.4

**Fig. 3:**
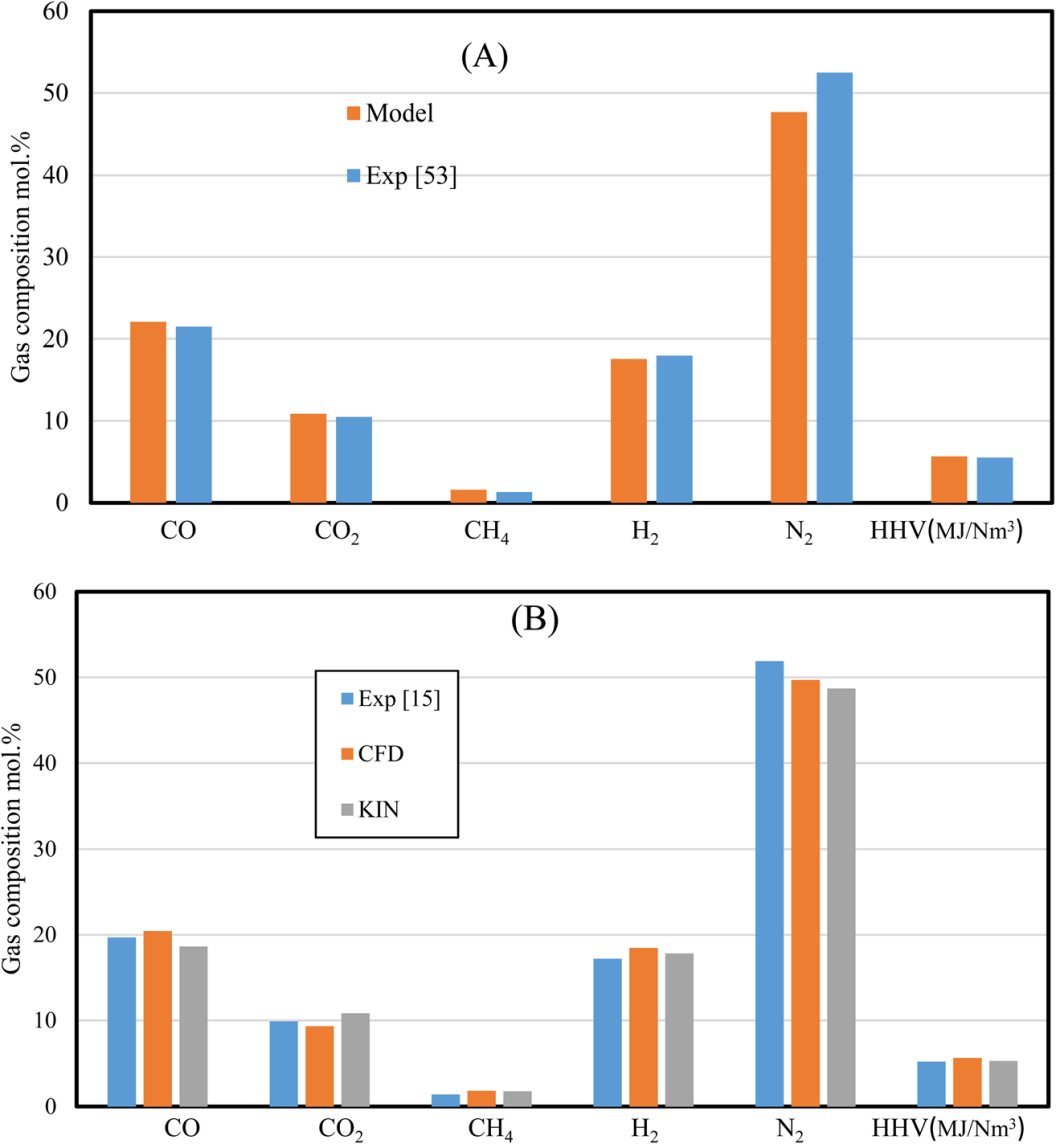
Current model validation for (A) wood pellets,^57^ and (B) rubber wood.^15^

The set of results illustrated by Fig. 3 shows the dry gas composition at the gasifier outlet for (A) wood chips, and (B) rubber wood gasification. The results are validated under the same working conditions (*i.e.*, MC 7.36%, ER 0.35, and gasifier design) for wood pellets. On the other hand, rubber wood gasification simulations are run under (MC 18.5%, and ER 0.326). The HHV variations for wood pellets and rubber wood are (<3%, and <7%) respectively, while other gas species are showing smaller variations. The model's ability to replicate the process of gasification in downdraft gasifiers is demonstrated by a satisfactory agreement between the current model, kinetic model, and the experimental data.

### 3.3. Air gasification

The effect of changing the throat ratio when using air as a gasification medium is investigated. The production of syngas, its heating value, velocity, and temperature distributions, as well as the composition of H_2_, CO, and CO_2_ at the producer is further illustrated.

#### 3.3.1. Throat diameter effect on air gasification process

Gasifier throat diameter is expected to affect the reactions and residence time inside the gasifier. As a result, it needs a careful consideration when designing a gasifier. A new dimensionless parameter, so called a throat ratio *r* is generated to simplify the procedure, where *r* is the ratio between the throat diameter and the gasifier diameter (also known as the fire box/pyrolysis diameter). Four different values for *r* will be used in the current study (0.4, 0.28, 0.23, and 0.14) to evaluate the effect of throat on the gasifier performance and syngas production.

#### 3.3.2. Temperature and velocity distributions

**Fig. 4:**
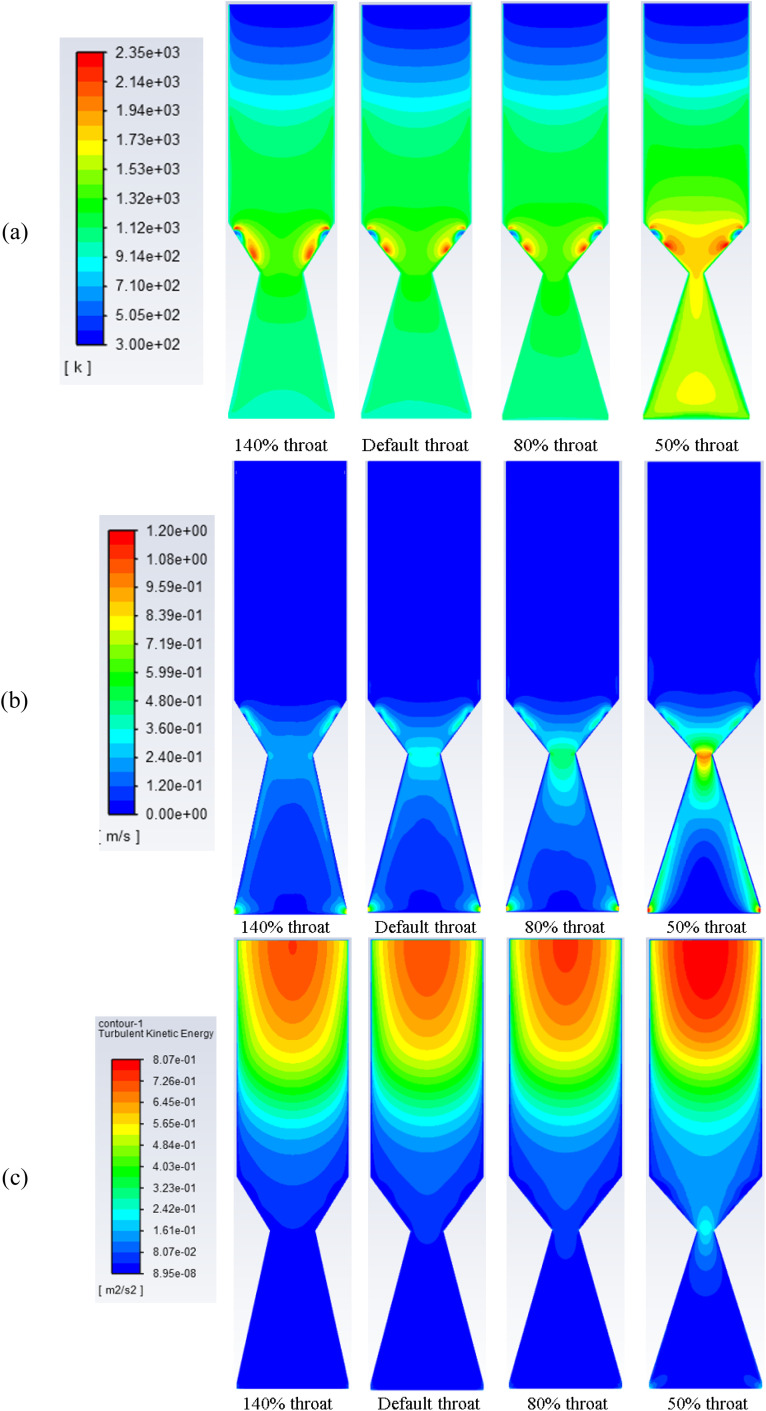
Contours of static temperature (a), velocity (b), and turbulent kinetic energy (c) along gasifier for air gasification at different throat diameters.

Fig. 4 illustrates the effect of changing throat ratio on the distribution of temperature (a), velocity (b), and turbulent kinetic energy (c) along the gasifier. Rubber wood is used with an ER of 0.3 and air as the gasifying medium. The default throat diameter based on the kinetic model^31^ predictions is 6.2 cm, and the gasifier diameter is 21.8 cm. Maximum temperatures around the nozzles (ignition temperature) are ∼2300 K, while at the centreline/centre zone of the gasifier ∼1650 K at the smallest throat ratio of 0.14 examined. For the design case, the maximum temperature along centreline is ∼1300 K which is in a good agreement with^55,62,63^ as well as the results derived from the kinetic model.^31^

Decreasing the throat diameter results in a gradual increase in the temperature inside the gasifier. This is clearly because of more throttling at the end of combustion zone which results in a longer residence time and higher turbulence (Fig. 4b), which in turn increasing the temperature. The volume of combustion zone has changed slightly because of the throttling effect. However, the model considers fixed flowrate of biomass and gasifying medium, which ensures the same flowrate inside the gasifier in all cases of changing throat size. As a result, when throat diameter is decreased, this led to an increase in turbulence, and residence time, and consequently, favours the oxidation reactions. Higher residence time and turbulence also encourage the combustion reactions (exothermic), leading to an increase in temperature and consumption of H_2_ which will be explored in more detail in the next sections. Also, as discussed that decreasing throat ratio leads to more turbulence inside the gasifier and within the combustion zone, which causes higher temperatures and velocity (Fig. 4b). Maximum velocity within the range of 1–1.2 m s^−1^ is achieved around the exit nozzles and at the throat area.

The set of results illustrated in Fig. 4c depicts the turbulence kinetic energy associated with air gasification at different throat ratios. The mean turbulent kinetic energy per unit mass generated during the gasification process shows higher values for the smallest throat diameters. More turbulence per unit mass starts at the pyrolysis then decrease along the gasifier height. As shown previously in Fig. 4c, higher velocities are formed around the air nozzles and the syngas exits. Additionally, for smaller throat ratios, higher turbulence and velocity are found. This is because of the higher residence time due to throttling and more ability for reactions to place. On the other hand, throttling generates higher velocities, and hence, higher turbulence.

#### 3.3.3. Producer gas composition and heating value

**Fig. 5:**
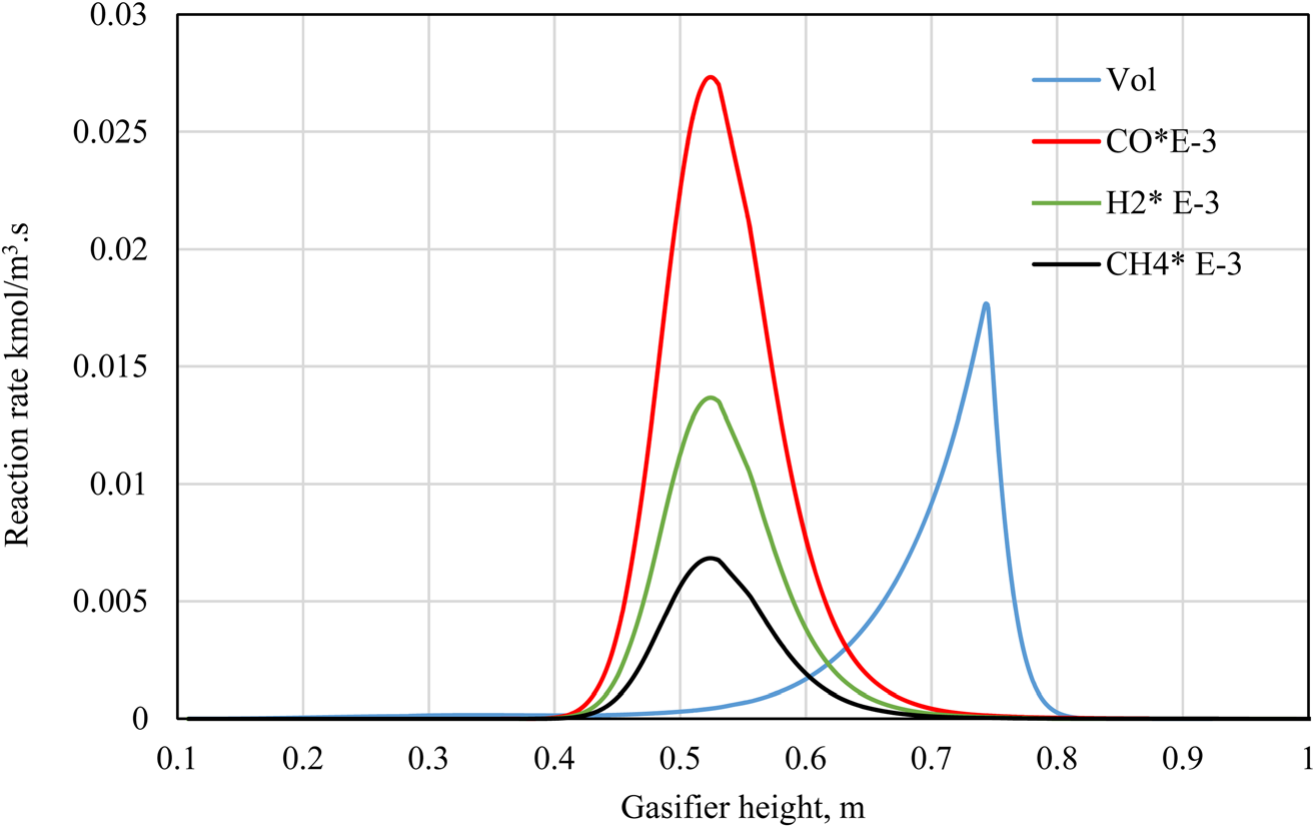
Volatiles decomposition and combustion reactions rate along gasifier.

As illustrated in Fig. 5, the volatile break-up process starts slightly below the top of the gasifier, *i.e.*, the pyrolysis zone. While at a height of 45 cm of the gasifier, all the volatiles tend to be fully decomposed and converted to other compounds in the combustion and gasification zones. The volatiles are converted into tar, char, and gases. The combustion rate of different gases is taking place at the combustion zone where it meets the oxidant (air) as illustrated clearly in the figure. The reaction rates in (kmol m^−3^ s^−1^) for CO, H_2_, and CH_4_ combustion for wood gasification at ER 0.3 is discussed. The combustion reactions take place between the gasifier heights of 40–60 cm. These reactions are exothermic, generating heat for the whole gasification process consisting of drying, pyrolysis decomposition, and gasification reactions. As a result, the combustion zone inside the gasifier has higher temperatures (Fig. 4). Higher reaction rates are found for CO, followed by H_2_, and CH_4_ respectively. This is because of increased activity of CO and H_2_, and thus larger amounts are produced during pyrolysis compared to CH_4_.

**Fig. 6:**
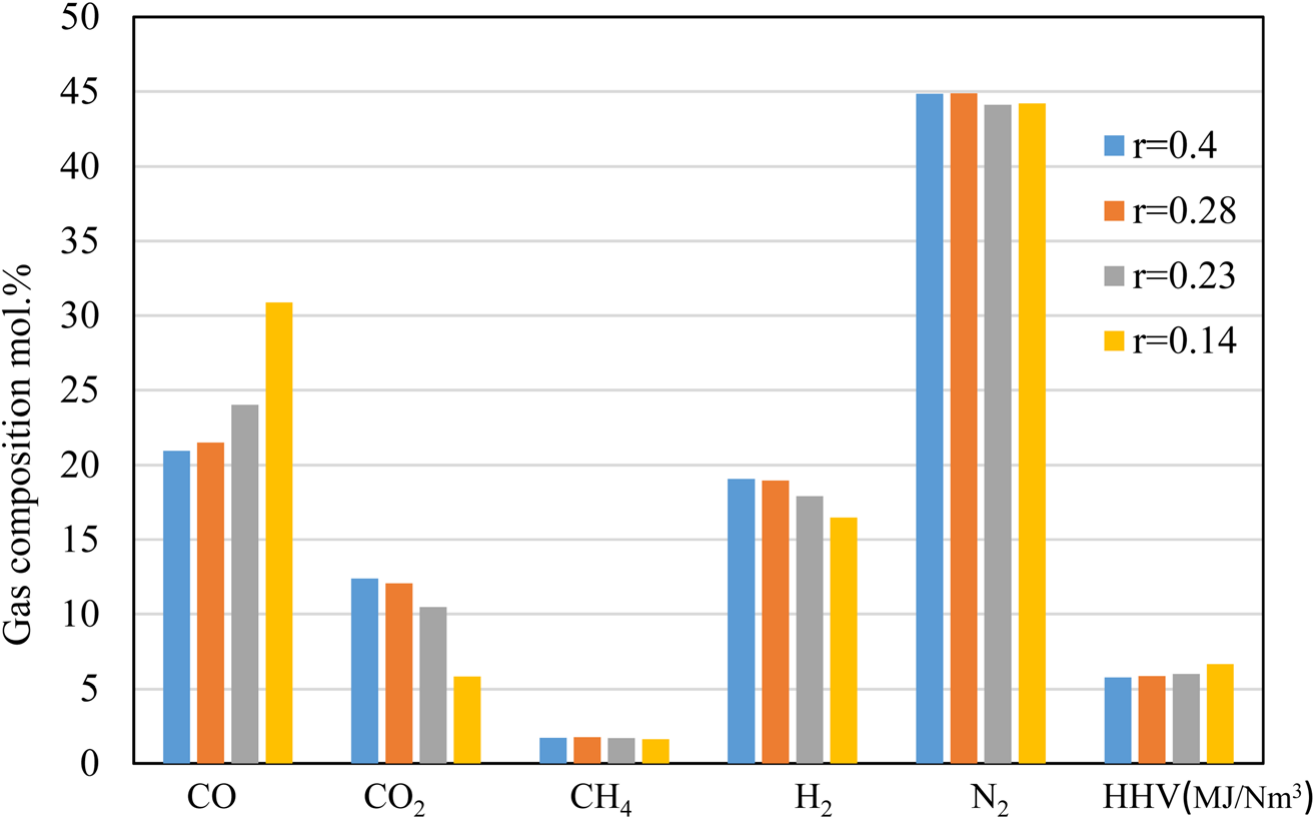
Producer gas compositions at different throat ratios (*r*) for air gasification.

The results shown in Fig. 6 depict the volumetric gas composition of the producer gas at different throat ratios. The throat ratio is set to *r* = 0.28 by default; however, increasing the throat does not significantly affect the producer syngas composition or heating value. In contrast, decreasing the throat diameter leads to an increase in the producer gas heating value. This is because a smaller throat diameter induces more throttling in the combustion area and increases residence time, which encourages heterogeneous combustion reactions (Fig. 5). This subsequently led to enhanced gasification process, resulting in an increase in CO, CH_4_. The boudouard, methanation and other reduction zone reactions are more likely to occur due to the rising temperature, resulting to consumption of CO_2_, and consequently, an increase in CO, and CH_4_, as shown in Fig. 6. Furthermore, the nitrogen concentration drops slightly, while the heating value tends to increase while reducing the throat ratio, again due to increase in the syngas composition. Optimum throat diameter is observed with highest values of CO, CH_4,_ and H_2_, and low CO_2_ concentrations (*i.e.*, the *r* = 0.14). As previously illustrated in Fig. 4, the smaller throat ratios lead to high residence time, and turbulence inside the gasifier. Consequently, more consumption for hydrogen as seen in Fig. 6. However, the decrease in H_2_ is ∼13% when using *r* = 0.14. On the other hand, there is increase in CO production by ∼43% when using *r* = 0.14 rather than default throat ratio (0.28). As a result, optimum throat diameters (*r* = 0.14) produce heating values ∼15% higher than other cases.

### 3.4. Oxy-gasification

#### 3.4.1. Temperature and velocity distributions

**Fig. 7:**
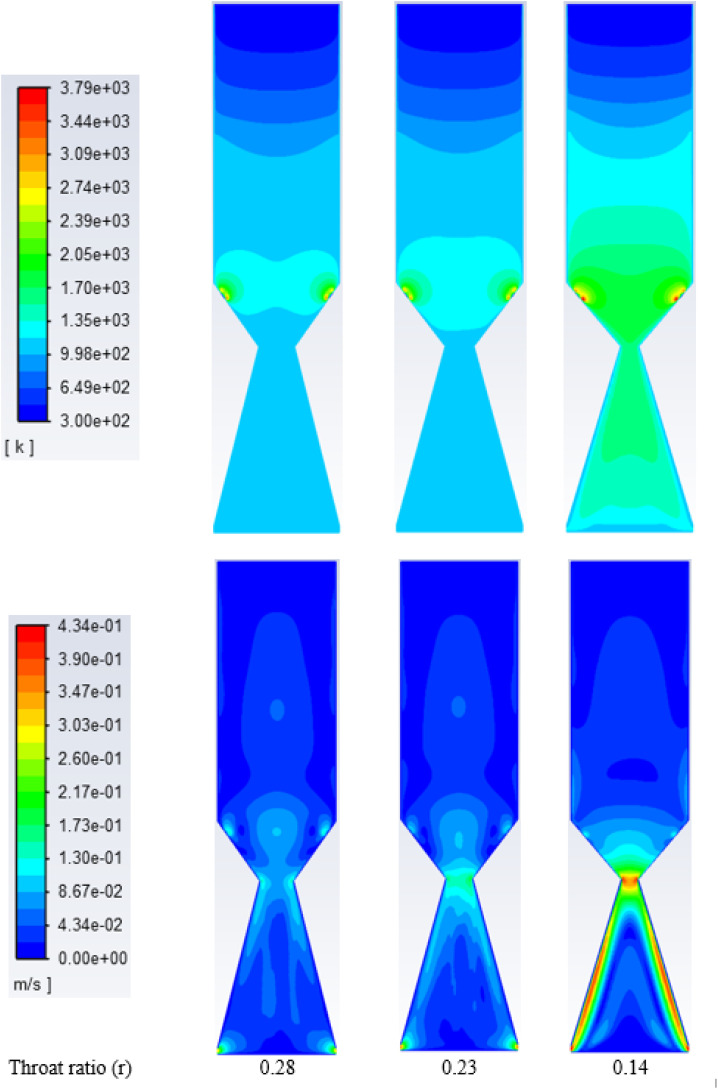
Contours of static temperature (top) and velocity along gasifier, (K) for oxygen at different throat diameters.

Fig. 7 depicts the temperature and velocity distribution along the gasifier when oxygen is used instead of air as the gasifying medium. Rubber wood is used at ER of 0.3, and an MC of 18.5%. All simulations are run under the same conditions for easier comparisons and optimum results. The temperature reached their highest level at 2400–3700 K near the oxygen injection points (nozzles). Temperature inside the gasifier rises while the throat diameter decreases, as expected, and already discussed with air gasification. It also exhibits temperature variations along the gasifier centreline from (1300–1700) K, and around 1050 K at the gasifier exit, which is consistent with experimental data in ref. 35. Furthermore, as previously discussed with air gasification, reducing throat leads to higher residence time, turbulence, and oxidation inside the gasifier, resulting in a temperature increase. Compared to air, oxy-gasification achieves higher temperatures because of the absence of nitrogen. As a result, fuel consumption is reduced, and higher flame temperature is achieved.

On the other hand, the velocity distribution inside the gasifier with oxy-gasification reaches a maximum of 0.4 m s^−1^, compared to 1.2 m s^−1^ with air gasification. As discussed earlier, for the same ER, a lower amount of oxygen is required to gasify the same amount of biomass. As a result, with the same throat diameter, smaller flow rates are achieved, resulting in lower velocities inside the gasifier.

#### 3.4.2. Producer gas composition

**Fig. 8:**
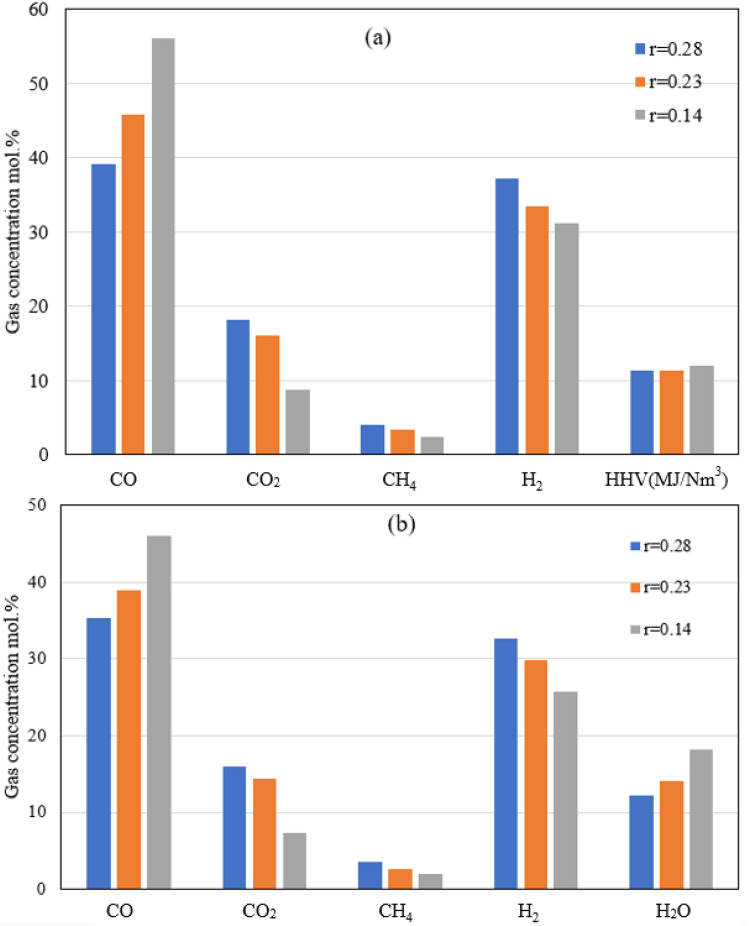
Producer gas volumetric composition (a: dry, and b: wet basis) at different throat ratios for oxy-gasification.

Fig. 8a illustrates the volumetric concentration of syngas species on a dry basis at the gasifier exit. In the absence of nitrogen, higher concentrations of syngas species are found, and hence resulting in a higher heating value for the producer gas. At the same working conditions of biomass, ER, and MC, the heating value is expected to be two times higher than that of air-gasification, which is in strong agreement with the results derived from previous research.^64–66^

Reduction in the throat ratio leads to an increase in the producer gas heating value. This is because of throttling, causing turbulence and higher temperature and residence time inside the gasifier, further leading to an increase in the gasification reaction rates with higher CO and lower CO_2_ concentrations. Higher concentrations of CO are due to increased rates of Boudouard reaction which consumes CO_2_ as noticed in the results. Slight differences in heating value were found while changing the throat ratio. The findings are matching with the same results from air gasification. Optimum throat ratio of (*r* = 0.14) leads to the higher production of CO, leading to increase the values of HHV to the maximum of 12.1 MJ Nm^−3^.

On the other hand, reversed steam reforming (CO_2_ + H_2_ CO + H_2_O) which has the highest activation energy, and pre-exponential factor (Table 3) leads to more consumption of H_2_ due to the higher temperatures (for lower throat ratios). As a result, lower H_2_ concentrations are found with low throat ratios. On the other hand, although higher temperature favours higher formation of CH_4_ through methanation and reforming reactions, CH_4_ concentration drops because of lower throat ratios (Fig. 6 and 8). This is further influenced by the higher reaction rates of reversed steam reforming and methane reforming reactions resulted in more CO with consumption of CH_4_. Additionally, this favours the formation of CO_2_. However, in the presence of char and higher temperatures, CO is formed through the boudouard reaction. Same effects are found during air and oxy-gasification. Additionally, the continuous consumption of H_2_, CH_4_ is also leading to H_2_O formation as illustrated by Fig. 8b referring to the abovementioned discussions and also as seen from the reactions at (Tables 2 and 3).

### 3.5. Towards CO_2_ free gasification

Sensitivity analysis is carried out to further study the effects of changing ER on both the syngas production (HHV) and CO_2_ emissions. Air and oxygen are used as gasifying medium while rubber wood is the feedstock. A fixed (the smallest) throat ratio (*r* = 0.14) is used because it proves to give higher heating values with lower CO_2_ production *e.g.*, see Fig. 6 and 8.

**Fig. 9:**
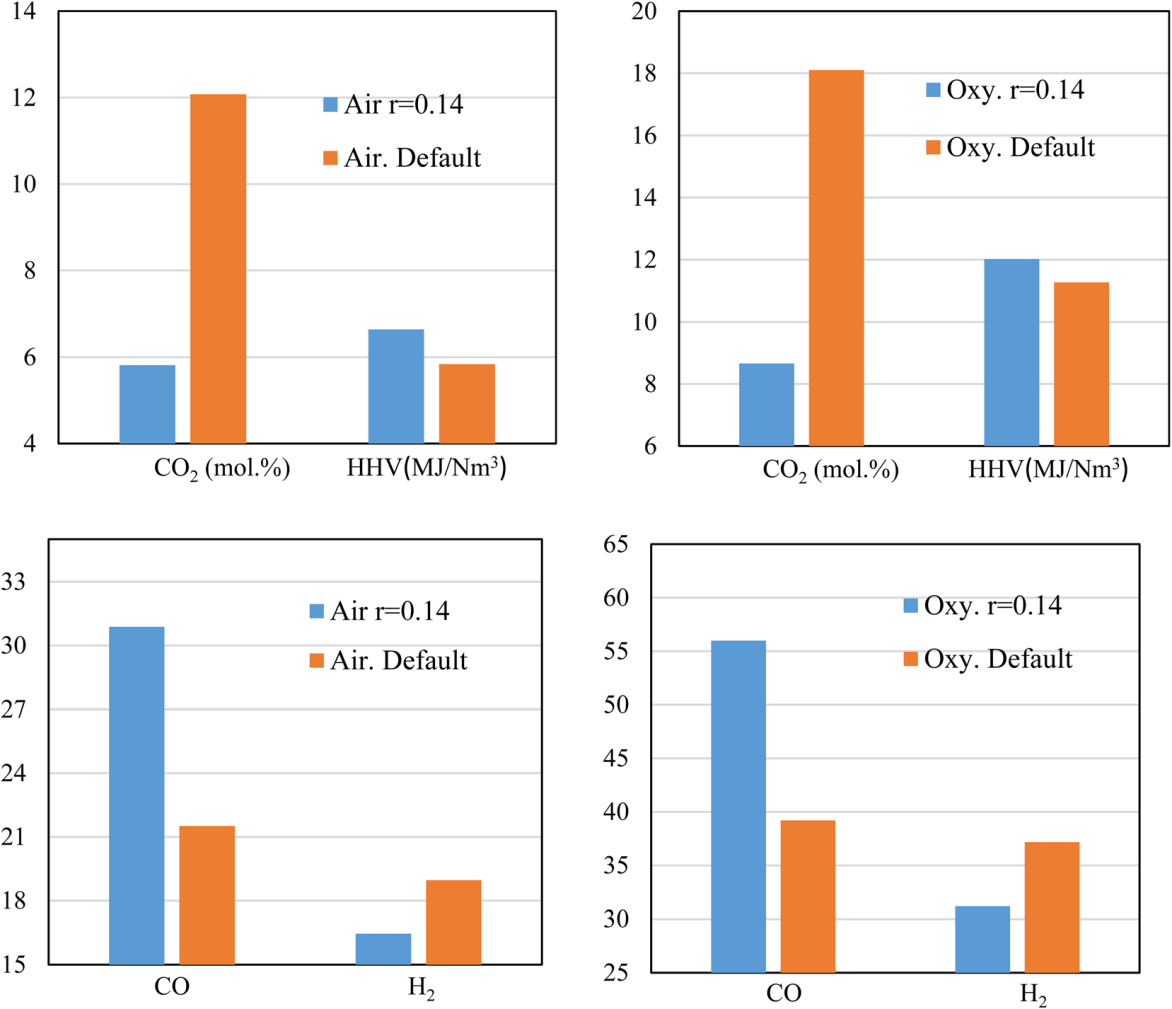
Effect of throat sizing on CO_2_, HHV, H_2_, and CO for air, and oxy-gasification.

Fig. 9 illustrates the effect of throat sizing on the H_2_, CO, CO_2_ produced during the gasification process, and the corresponding heating value, where the default value of throat ratio *r* = 0.28. For air, and oxy-gasification, throat ratio of (*r* = 0.14) leads to (∼52%) reduction in CO_2_ production. The reduction in CO_2_ amount is because of the previous discussions showing that small throat leads higher temperatures, higher residence time, and hence encourage the heterogenous reactions to take place (Fig. 4, 5, and 7). As a result, the methanation, and boudouard reactions are taking place and consuming more CO_2_. Thus, higher CO production is also achieved resulting in increasing the heating value of producer gas. For a throat ratio of 0.14, the heating value was found to increase by ∼ (6–14%) than default throat ratio. Hence, throat sizing seems to be a very promising option for eliminating CO_2_ emissions within the process gasification. Although the study was aiming to produce CO_2_-free syngas, the massive reduction in the produced values (*i.e.*, ∼52% reduction) without the further use of solvents, catalysts, or another means of CO_2_ capture is encouraging and offers a major improvement in the gasification process.

While reducing the throat ratio, the results in both the cases (air and oxygen) follow the same behaviour of increasing CO, and a decrease of H_2_. An increase of CO production was found to be up to 43% for both air and oxy-gasification, while for the same throat decrease, the H_2_ values are found to drop by (15–19%). As previously discussed, one of the main aims of the current study is the decrease of CO_2_. As a result, an increase in CO was found, because of the continuous use of CO_2_ in the boudouard and the methanation reactions. Also, H_2_ is consumed because of higher residence time and in the presence of CO_2_ to be further converted into CO (CO_2_ + H_2_ → CO + H_2_O). Consequently, this affects the concentration of other species leading to decrease of H_2_. Although H_2_ is decreasing, the increase in CO leads to a higher increase in the heating value of the produced gas. This is due to the fact that the ratio of CO increase is higher than H_2_ reduction, since it relies on CO_2_ consumption as previously shown in Fig. 9.

**Fig. 10:**
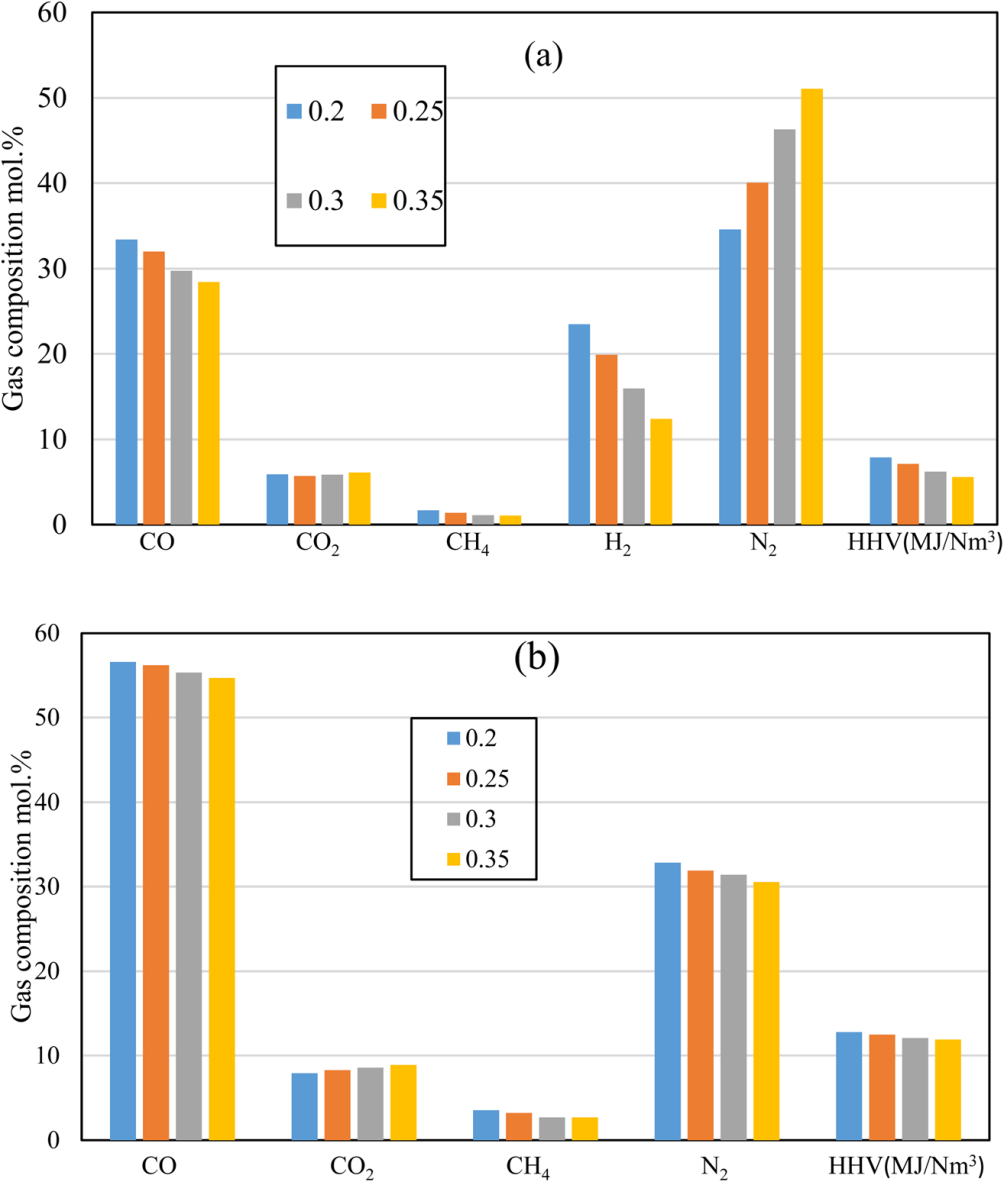
Effect of changing ER on syngas production for (a) air, and (b) oxy-gasification.

Fig. 10 illustrates the producer gas composition at different ER for the air, and oxy gasification at the same working conditions. Rubber wood is used as feedstock at ER of 0.2, 0.25, 0.3, and 0.35, for the same throat ratio (0.14). One of main aims of the current study is reducing/eliminating the production of N_2_, and CO_2_. As shown in the figure, air gasification produces higher amounts of N_2_ (40–45) mol% because of its higher nitrogen content. On the other hand, oxy-gasification shows zero content of N_2_. This is clearly because it does not have any content of N_2_. Throat ratio change has no effect on N_2_ production because it only changes with the amount of air injected (*i.e.*, the equivalence ratio) as seen in Fig. 6.

While varying the throat ratio, the amounts of CO_2_ production show similar amounts for both air and oxygen. However, it shows small amounts of CO_2_ during air gasification (CO_2_ ∼ 5.7–6 mol%). This is mainly due to the throttling which tends to increase the residence time inside the gasifier, temperature (Fig. 7) and gives the opportunity to boudouard reaction to take place, and more CO_2_ consumption.

Fig. 10b also shows the same effect of CO_2_ reduction while reducing the ER and using smaller throat ratio. However, oxygen tends to produce more CO_2_ than air gasification for the same working parameters (ER, Feedstock, and throat ratio). Nitrogen free gasifying mediums (oxygen) tends to produce higher concentrations of other components. As a result, higher CO_2_ production than air gasification. Additionally, slight changes in all gas composition and the corresponding heating value were reported in this case (*r* = 0.14), irrespective to the change of ER. For the same ER, the change of *r* from 0.28 to 0.14 results in increase in CO and HHV by 41% and 8% respectively, while reducing CO_2_ and H_2_ concentrations by 53% and 16% respectively. This in general tends to increase HHV, though H_2_ concentration is decreasing. As a result, the throat change has an effect on increasing syngas heating value and reducing CO_2_ emissions. Lower ER tends to produce more CO, H_2_, CH_4_, resulting in higher HHV. However, particular to note for the lower throat ratio of 0.14 that ER effect is found to be small (Fig. 10b). This is because of the throttling effect which consumes higher amounts of CO_2_, H_2_, and CH_4_ resulting in higher production of CO as previously illustrated in Fig. 6 and 8. Nevertheless, this effect was not clear in air gasification because of the nitrogen dilution in the gasifying medium. However, in oxy-gasification, since the optimal condition was achieved at *r* = 0.14, the maximum production of CO with lowest amounts of CO_2_ was achieved (regardless of ER change). Moreover, lower throat ratio is associated with higher combustion and gasification temperatures, and reaction rates (Fig. 7) even at lower ER, which favours the CO formation and results in HHV increase as ER increases from 0.2 to 0.35 and results in decrease of CO,H_2_, and HHV by 3.5%, 7.5%, and 7.3% respectively. Simultaneously, this results in CO_2_ reduction by 11%.

The research also aims to increase the amounts of H_2_ and CH_4_ which in turn increase the heating value as shown in the figure. Lower heating values with lower syngas composition is noted for air compared to oxygen gasification. This is because of the N_2_ dilution in air gasification (∼50%). On the other hand, oxygen tends to increase the production of CO, H_2_, and CH_4_ as shown in the figure. The smallest throat ratio, with lower ER of 0.2, leads to the highest amounts produced from CO, H_2_, and CH_4_ which increase the heating value to the maximum 12.7 MJ Nm^−3^. As discussed earlier, decreasing the throat ratio, leads to higher residence time, higher temperature, better mixing, and turbulence. All the previous mentioned factors lead to higher production of CO, H_2_, and CH_4_ which further increases the heating value. Furthermore, the highest heating value in the current work is obviously higher than previous works using oxy-gasification *e.g.* ref. 67 (10.1 MJ Nm^−3^), ref. 41 (10.12 MJ Nm^−3^) and ref. 61 (11 MJ Nm^−3^). This is because of the effect of throat ratio on the gasification process.

### 3.6. Producer gas yield, and gasification efficiency

The throat diameter change has a great impact on the producer gas quality (Fig. 6, 8, and 10) including gas composition, and the corresponding heating value for air and oxy-gasification. However, a full understanding of the process should include the yield of produced gas and the gasification efficiency for full understanding of the whole process. Gasification efficiency is calculated as follows:^38^9
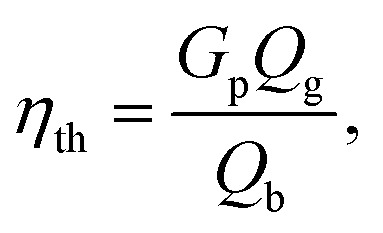
where *Q*_g_ is the syngas LHV in (MJ Nm^−3^), *G*_p_ is the produced gas yield in Nm^3^ kg^−1^, and *Q*_b_ is the biomass LHV in MJ kg^−1^ and estimated as following.^68^

10 *Q*_b_ = 0.339 C + 1.029 H + 0.109 S − 0.112 O − 0.025 W

11 *Q*_g_ = 0.126 CO + 0.108 H_2_ + 0.358 CH_4_

where C, H, O, S are the elemental composition of the feedstock, and W is the moisture content. While CO, H_2_, and CH_4_ are the volume fraction of different species in the producer gas.

**Fig. 11:**
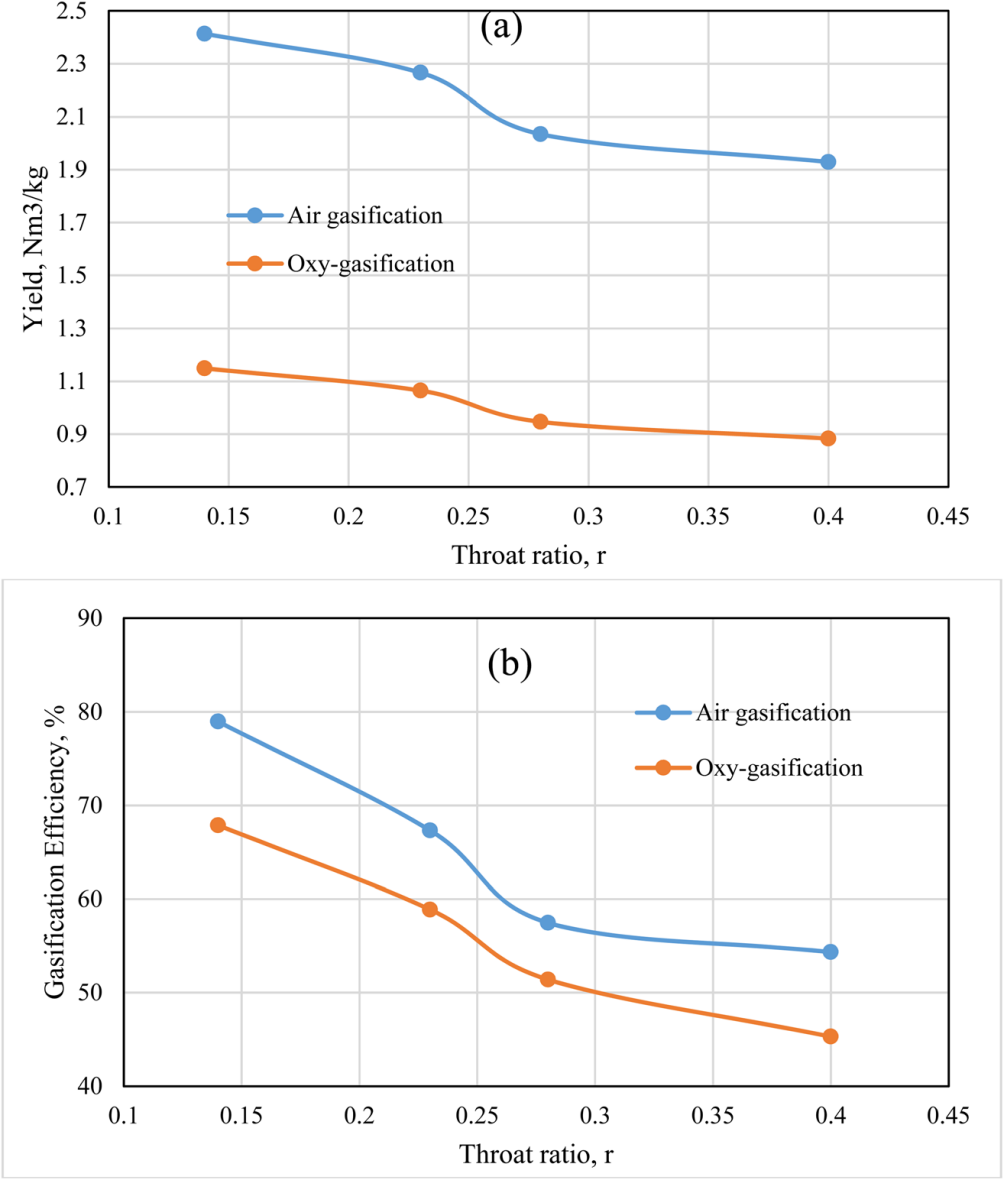
Producer gas yield (a), and gasification efficiency (b) for air and oxy-gasification.

The results illustrated by Fig. 11 depict the effect of changing throat ratio on the producer gas yield, and the gasification efficiency for rubber wood at fixed ER = 0.3, and MC 18.5%. Under a certain ER, the model uses fixed flowrate of biomass and gasifying medium no matter the throat ratio changes, resulting in the same flowrate for all cases. However, the throat ratio changes lead to a change in temperature, velocity, and different gas species concentrations, and the corresponding heating value of the produced gas (Fig. 4, 6, 7, and 8). The aforementioned factors are all affecting the yield of produced gas as illustrated by Fig. 11. Air has higher yield than oxy-gasification – although same ER – nitrogen content in the air tends to feed higher amounts of air than oxygen as a feeding medium for the same working conditions. As a result, this tends to increase the gasification efficiency for the same feedstock (eqn (11)).

Lower throat ratios tend to produce higher velocities, temperatures, and heating values for produced gas as previously illustrated. As a result, this effect leads to higher velocities near the exit of the gasifier, and volume flowrate for the producer gas, and correspondingly higher yield. On the other hand, lower throat ratios are found to produce higher syngas composition, which in turn favours higher heating values resulting in higher gasification efficiencies. As previously suggested in Fig. 6 and 8, and in the current figure, the optimum throat ratio is *r* = 0.14. At *r* = 0.14, the gasification efficiency increased that the base design case (*r* = 0.28) by 32, 37% for oxy, and air gasification respectively. While the producer gas yield is found to increase at the optimum throat ratio than the base case by 22, 19% for oxy, and air gasification respectively. Air and oxy-gasification producer gas yield are ranging between (1.9–2.4), and (0.88–1.1) Nm^3^ kg^−1^ of biomass respectively. Additionally, the gasification efficiency ranges between (54–79)%, and (45–68)% for air and oxy-gasification respectively. The results meet fair agreement with literature data of ref. 68, 69, and 70.

### 3.7. Carbon conversion

Carbon is the main component during the process gasification. As a result, the carbon conversion from the biomass to the product gas is represented by carbon conversion efficiency *η*_cc_. Carbon conversion efficiency is the proportion of converted carbon into gases (in producer gas) to the total amount of carbon in the feedstock and is estimated from ref. 71 and 72 as following.12
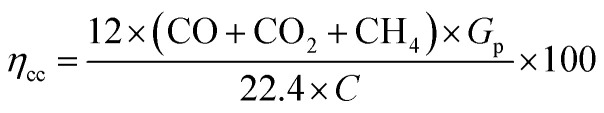
where CO, CO_2_, CH_4_ are the volume concentrations of different species in the producer gas, *C* is the carbon concentration in the feedstock, and *G*_p_ is the yield of producer gas.

**Fig. 12:**
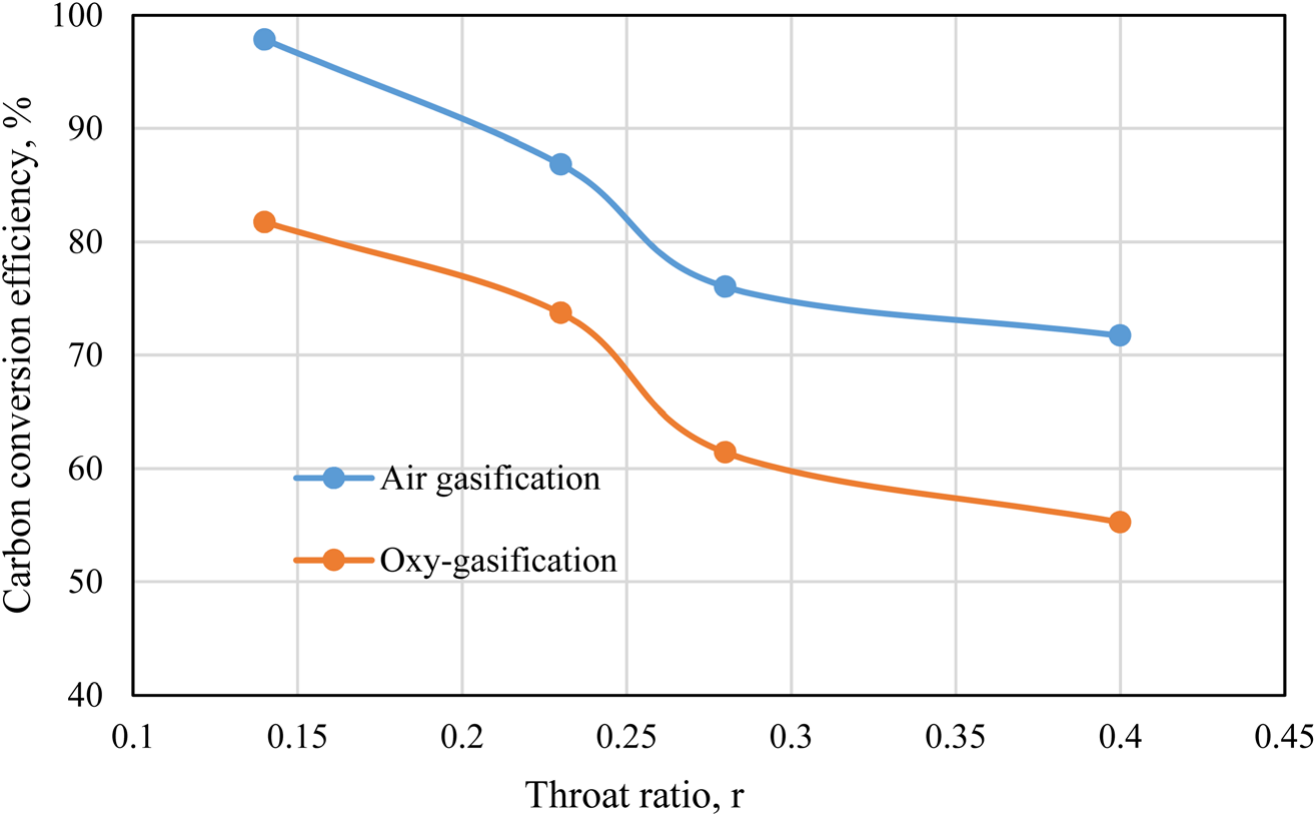
Carbon conversion efficiency for air and oxy-gasification.

Fig. 12 represents the carbon conversion efficiency during rubber wood gasification. Air and oxygen are used as gasifying mediums under the same working conditions of ER = 0.3, and MC = 18.5%. Fixed working parameters are used for easier comparison between air and oxy-gasification, and during throat ratio change. Air yields higher conversion efficiencies than oxygen under all cases. Although carbon fraction in producer gas (CO + CO_2_ + CH_4_) is higher during oxy-gasification, but the yield of producer gas during air gasification is more than double oxy-gasification during same conditions (Fig. 11). As a result, this tends to increase the conversion of carbon during air gasification.

Lower throat ratios are associated with higher amount of carbon fraction in producer gas (Fig. 6 and 8) and higher yield of syngas, resulting in higher carbon conversion than higher throat ratios. The carbon conversion during air and oxy-gasification is ranging between (71–98), and (55–82)% respectively. For the optimum throat ratio, carbon conversion is higher than the design/base case by 28.8, and 33% for air, and oxy-gasification respectively. This finds a strong agreement with previous works of ref. 71, 72, 73.

The unit cost of natural gas was reported to be around 1–3 US$ per GJ.^74,75^ On the other hand, for the syngas produced by oxy-gasification, the unit cost is estimated to be 2.0 US$ per GJ. However, this requires a detailed economic study to evaluate the exact cost of the syngas based on feedstock, gasifying agent, technology, and maintenance. As a result, lower throat ratios are effective in reducing CO_2_ emissions, boosting gasifier performance, increasing syngas yield, HHV, gasification efficiency, and achieves higher carbon conversion during the process gasification. The gasifier model is based on a 20 kW downdraft biomass gasifier (small industrial scale). However, the results derived from the model are applicable in both small and large industrial scales. The dry gas composition results are based on specific working conditions (ER, MC, feedstock) regardless of gasifier scale (Fig. 6 and 8). Additionally, the results represented in (Fig. 11, and 12) for gasifier performance are independent of the gasifier capacity since gas yield (Nm^3^ per kg of biomass), and the efficiencies in %. As a result, the findings represented by the current research could be applied in different scales of gasifiers and for multiple applications.

## 4. Conclusions

A CFD model was developed to investigate the effects of varying gasifying mediums and throat ratios on the gasification process performance. Producer gas composition, heating value, CO_2_, N_2_, temperature, and velocity distributions were presented and discussed. The model is validated through mesh independency test, and then against results derived from experiment for the same gasifier type, dimensions, feedstock, and working conditions.

The results revealed higher heating value for oxy-gasification than air gasification. Additionally, 4 throat ratios were examined in the current study (0.14, 0.23, 0.28, and 0.4) and lower throat ratios tend to increase the producer gas heating value, and temperature along the gasifier. Lower throat ratios are also preferred when it comes to reducing CO_2_ amounts for air gasification. Furthermore, the lowest throat ratio resulted in a CO_2_ reduction of more than 55% and a 20% increase in HHV, as compared to the default cases used in previous designs. Furthermore, lowest throat ratio yields higher production of producer gas, gasification, and carbon conversion efficiency by 22, 37, and 33% respectively. As a result, the current study gives promising outcomes in reducing CO_2_ and N_2_ emissions in the gasification process without the need of using any filters, removal, or catalysts. Additionally, the change in design/throat sizing is applicable in any downdraft or updraft system and independent on gasifier size/capacity.

## Nomenclature

### Upper case letters

*A*
: Pre-exponential factor, (units vary)
*D*
: Diameter (m)
*D*_*i*,m_
: Mass diffusion coefficient for species *i* in the mixture
*D*_T,*i*_
: Thermal diffusion coefficient for species *i*
*D*_t_
: Turbulent diffusivity
E: Energy, (kJ mol^−1^)
*F*_*i*_
: External body forces, (N)
*G*_b_
: Turbulence kinetic energy due to buoyancy
*G*_k_
: Turbulence kinetic energy due to the mean velocity gradients
*H*
: Enthalpy, (kJ mol^−1^)
*I*
: Unit tensor
*J*_*i*_
: Diffusion flux of species *i*
*K*
: Kinetic constant, (s^−1^)
*M*
: Molecular mass, (kg mol^−1^)
*P*
: Pressure, (Pa)
*R*
: Net rate of formation, (mol m^−3^ s^−1^)
Re: Reynolds number
*R*_*i*_
: Net rate of production of species *i* by chemical reaction
*S*_k_
: Source terms for the kinetic energy
*S*_t_
: Mass added to the continuous phase from the dispersed phase
*S*_*ε*_
: Source terms for rate of dissipation
Sc_t_: Schmidt number for turbulent flow
*T*
: Temperature, (K)
*T*_R_
: Temperature of radiation (K)
*V*
: Volume (m^3^)
*Y*_*i*_
: Mass fraction of species *i*
*Y*_M_
: Contribution of the fluctuating dilatation in compressible turbulence to the overall dissipation rate

### Lower case letters

*g*_*i*_
: Gravitational body forces
h
: Convective heat transfer coefficient (W m^−2^ K)
*h*_fg_
: Latent heat (J kg^−1^)
*m*_p_
: Mass of the particle (kg)
*x*_*i*_
: Number of mole species

### Greek letters

*ρ*
: Density
∑: Summation
Δ: Change in state
*τ*_*i*,*j*_
: Stress tensor
*μ*
: Molecular viscosity
*σ*_*k*_
: Turbulent Prandtl numbers for *k*
*σ*_*ε*_
: Turbulent Prandtl numbers for *ε*
*μ*_t_
: Turbulent viscosity
*ρ*_p_
: Density of the particle
*ε*_p_
: Particle emissivity
*σ*
: Stefan Boltzmann constant, 
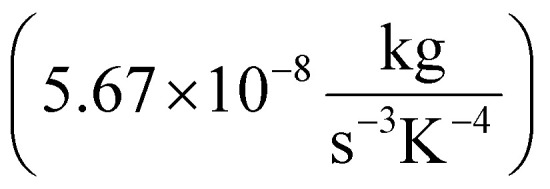

### List of acronyms

VOF: Volume of fluid
MC: Moisture content, (%)
A/F: Air to fuel ratio
ER: Equivalence ratio
HHV: Higher heating value (MJ Nm^−3^)
Nm^3^: Normal cubic meter
CFD: Computational Fluid Dynamics
DPM: Discrete phase model
PRESTO: PREssure Staggering Option
RANS: Reynolds Averaged Navier–Stokes

## Conflicts of interest

There are no conflicts to declare.

## Supplementary Material
